# Expecting better: a leadership reflection on loss, inequity and the future of maternal health

**DOI:** 10.1136/leader-2025-001501

**Published:** 2026-03-04

**Authors:** Sian Reece

**Affiliations:** University of Exeter, Exeter, UK

## Abstract

**Objective:**

To use a lived-experience account of stillbirth and subsequent pregnancy to examine how maternity care systems respond to trauma, reveal structural and relational failures in maternal health services and propose principles for more just, compassionate leadership in maternity care.

**Background:**

Despite advances in clinical knowledge, maternal outcomes in the UK have plateaued, with widening inequities by ethnicity, deprivation and other intersecting forms of exclusion. Maternal trauma, including stillbirth and miscarriage, is frequently minimised, poorly recognised in policy and measurement frameworks and inadequately supported in routine care. Personal experience of stillbirth and subsequent pregnancy exposes how systems that appear evidence-based and guideline-driven can fail to provide continuity, psychological safety and trauma-informed support at women’s most vulnerable moments.

**Methods:**

This reflective paper integrates first-person narrative of stillbirth, subsequent pregnancy and encounters with maternity and primary care services with professional insight from clinical and leadership roles. The reflection is informed by existing evidence on maternal outcomes, perinatal mental health, intersectionality and global trends in women’s health and rights. The narrative is used as an analytic lens to explore how leadership, culture and structures in maternity care shape women’s experiences and outcomes.

**Results:**

The reflection identifies recurrent gaps: silencing of women’s voices, inadequate bereavement and mental health support, fragmentation of care and limited recognition of trauma across the maternity pathway. It highlights structural injustice in maternal health, including inequities by race, poverty and migration status, and the marginalisation of outcomes such as stillbirth and early pregnancy loss in key indicators. It proposes leadership practices that are relational, trauma-informed and equity-focused, including embedding lived experience in governance, investing across the preconception-to-postpartum continuum and prioritising culturally safe, psychologically safe care.

**Conclusion:**

Leadership in maternity care must move beyond metrics and guidelines towards models grounded in humility, listening and justice. By centring lived experience, recognising trauma and addressing structural inequities, leaders can begin to rebuild trust, honour loss and reshape maternity systems to better serve women, babies, families and future generations.

My experience with ultrasound scans was limited but sufficient to understand the terrible truth laid bare on the screen beside us. There, in black and white, was the delicate image of our daughter’s heart; four chambers and two sets of valves, perfectly formed and completely motionless. Horror tightened its grip around me as the doctor moved swiftly, capturing the intricate details of her heart from every possible angle.

Tears flooded my eyes, blurring my vision, turning the screen into a distorted haze. The knowledge it held was unbearable. The doctor worked in silence, his concentration unwavering while I lay paralysed by fear. My husband remained by my side, his hand wrapped around mine, a lifeline of hope he refused to relinquish, even as mine slipped away.

The tiny cubicle was filled with the hushed presence of midwives, their bodies pressed close, their faces sombre. Desperation twisted my thoughts, pushing all reason aside. I convinced myself, with deluded optimism, they had assembled for an emergency delivery. An optimism that meant my ultrasound skills were lacking and our baby safe, other than the need for urgent delivery.

I glanced from one midwife to another, their eyes reflecting the devastating truth I was too terrified to accept. The crushing silence bore down on me. One midwife cried as she reached out to hold my empty hand. The doctor removed the ultrasound probe and turned to face us. The doctor’s words, when they came, were hesitant, as if he was choosing each one with care.

“I am so terribly sorry,” he began, his voice trembling, barely above a whisper. “I cannot find a heartbeat. Your baby has died.”

I did not plan to become a voice in this space. Like so many women, I assumed that when I needed the health system most, it would be there for me. As a doctor, I knew the statistics and I trusted the guidelines. I trusted the healthcare system that had become an intrinsic part of my identity. I lived and breathed the values of our healthcare system and the people who cared and were cared for.

That reality shattered on my due date when my daughter was stillborn. It was not only the death of my daughter that devastated my world, but the haunting silence that followed. We were completely lost. I found myself grieving not only my child but also the version of maternity care I had believed, had trusted, existed.

I had expected continuity, compassion and clarity. I thought there would be a plan or process. There would be someone to guide us, hold space for us and help us navigate the shock and sorrow of losing a child. I thought there would be someone who knew what to say, or at least who would not turn away in silence. When we were not offered compassionate care or access to mental health support for ourselves or our family, I at least expected to receive standard postnatal care but I was not even offered a postnatal check by my General Practitioner (GP). We were simply left in silence.

Soon after our loss, I became pregnant again. But my subsequent pregnancy revealed, with even greater clarity, how unprepared the system was to provide the compassionate, trauma-informed care we so desperately needed. Instead of feeling supported, my trauma, a constant source of irritation, an afterthought at best, intensified and became compounded. When I shared how anxious I was and asked for a referral to perinatal mental health services, my referral was rejected stating my current care was sufficient to meet my needs. There was no further discussion, no exploration of what I was experiencing and certainly no support offered.

I saw how systems fail women, especially at their most vulnerable. I experienced how trauma is minimised or misunderstood in clinical care, and I witnessed how some voices are consistently dismissed or ignored.

From the rollback of women’s rights globally to the persistent underinvestment in maternal services here in the UK, these are issues we can no longer afford to treat as niche.^1–4^ Maternal health is not just about the health of mothers. It is about children, families and the long-term well-being of future generations. The first 1001 days of life, from conception to age two,^5^ are foundational. How we support women during that time matters more than we often acknowledge. Preconception care must also be recognised as a critical part of the maternal health journey.^6^ Supporting women’s health before pregnancy through nutrition, mental health, chronic disease management and reproductive planning is essential to delivering the best outcomes for both mothers and babies.

This reflection is written from a place of both professional insight and personal loss and is a call for leadership to address the rising crisis in maternity care. In the following sections, I explore what maternity care reveals about our health systems and about our society as a whole, why women’s health continues to be deprioritised, and how we might begin to lead with courage, justice and compassion. It is a tribute to the many women who have been failed by systems not built for them and it is a hopeful offering that we can, and must, do better.

## The Injustice of Maternal Health

If you want to understand how a society values women, look at how it treats those giving birth. The truth is often unspoken, hiding between the statistics and the policy statements, but it is there. I had seen the reports and numbers for years, but only after my own experience did I truly feel the injustice they represent.

In the UK, maternal outcomes have plateaued and for some groups, they are worsening, despite advances in clinical knowledge, technology and public health. Black women are twice as likely to die from pregnancy or childbirth than white women, and women in the poorest parts of the country face significantly higher risks, during and after pregnancy, than those in wealthier areas.^7^ These are not just isolated data points; they are evidence of structural injustice.

Across the world, we are witnessing an erosion of women’s rights and health autonomy that directly impacts maternity care. In the USA, the overturning of Roe v. Wade has led to widespread restrictions in reproductive care, with early evidence of rising maternal mortality and dangerous delays in emergency obstetric treatment. In Afghanistan, the stripping away of women’s basic rights to education, employment and healthcare has already caused a catastrophic decline in access to maternal health.^8^ Recent internet and phone blackouts have further isolated women and healthcare providers, compounding the humanitarian crisis and making it even harder to reach those in need.^9^ And in parts of sub-Saharan Africa, shortages of skilled birth attendants and essential medical supplies continue to make childbirth perilous. These trends are not abstract; they are the lived reality for millions, revealing how deeply gender inequity is embedded in health systems worldwide.

Maternal health is not just about clinical guidelines or care pathways. It is about dignity, trust and autonomy. The state of maternity care reflects how systems operate under pressure and who is protected when resources are scarce. We must not only confront harm retrospectively but act preventatively to protect the health and well-being of mothers and future generations.

The announcement of a national maternity investigation in England has brought renewed attention to the depth of the problem.^10^ Acknowledging harm across more than 15 years of service should prompt not only reflection, but radical reform. When we neglect maternal care, it is not only the women who suffer. Children suffer, families suffer and future health suffers.

Despite this, funding for maternal health services and research for maternal health remains disproportionately low. Investment lags behind both need and impact.^11–13^ Midwives are leaving the profession in alarming numbers.^13^ Maternity units are understaffed and care is fragmented.^14^ Meanwhile, opportunities to innovate or learn from lived experiences are too often missed.

To lead in this space is to stand at the centre of one of the most pressing public health, social justice and human rights issues of our time. The question is not whether we can afford to prioritise maternity care, the question is how we have ever justified not doing so.

### The unheard majority

You cannot nurture the next generation if you abandon the women carrying it. Too many women enter pregnancy expecting to be heard and leave feeling invisible. Their pain is dismissed, their symptoms minimised. Their trauma is ignored or medicalised without meaning. For women with one or more systemically excluded identities, such as race, disability or migration status, the risk of not being heard is not just distressing, it can be fatal. These overlapping forms of exclusion are not simply additive; they interact in complex ways, compounding risk and widening inequity, an approach captured by the lens of intersectionality.

There is no shortage of data. Globally, the silencing of women in maternity care is widespread. In India, studies have shown that nearly one in three women experiences mistreatment or abuse during facility-based childbirth.^15^ In Nigeria, many women report being denied dignity, pain relief or informed consent during labour.^16^ In countries such as Canada and Australia, Indigenous women continue to face higher rates of stillbirth and maternal complications due to systemic racism and disrespectful care.^17^

We have report after report showing disparities in maternal health outcomes.^18^ We know that perinatal mental illness is a leading cause of maternal death in the UK. Yet, the translation of this knowledge into action is painfully slow. Services remain stretched and uneven. The research that could shape future care is underfunded and often disconnected from lived reality.

At the heart of the issue is a more uncomfortable truth; many health systems do not trust women’s accounts of their own bodies and they especially do not trust those who speak with pain, anger or complexity. The result is a culture of silencing.

When we ignore maternal trauma, we do not just harm women, we harm their children.^19^ The first 1001 days of life, beginning in pregnancy and lasting until the age of two, are critical for brain development, attachment and lifelong well-being. If a mother’s health is compromised, so too is the environment that shapes her child’s earliest experiences. Yet, we continue to treat maternal and child health as separate, as though care for one has nothing to do with the other. Leadership must move beyond these divides.

### The language of compassion and the art of listening

Leadership in maternity care must be relational, responsive and rooted in justice. It begins with listening, not just to statistics, but to people. My own loss also transformed how I communicate. I understand that compassionate, open conversation is not a luxury in healthcare, but a necessity. Now, when I sit with a person who has suffered trauma or grief, I listen more deeply and I acknowledge their pain instead of sweeping it aside.

Bereavement care guidelines emphasise shared decision-making after stillbirth, recognising that it can improve women’s long-term health and well-being. I learnt that what is needed most from our healthcare system is not a rigid adherence to clinical guidelines, but the courage to speak of the unspeakable, to step into the silence and to consciously care without flinching.

There is power in diverse storytelling. Art, oral testimony, photography and creative expression open space for truths that statistics and guidelines alone cannot reach. These forms foster empathy and solidarity. For me, I found comfort in writing of my experiences, to find the words to bring understanding and meaning to the trauma we are still learning to reconcile with the world. There was also photography, when words alone could not suffice. Leadership must make space for many forms of knowledge, including the emotional and creative. As Dr David Fakunle notes, storytelling is medicine.^20^ It heals systems as well as people.

### Leading what comes next

Maternal health is not a silo, it is the soil from which the future grows. We must lead like it matters, because it does. We cannot keep leading in ways that prioritise metrics without meaning. Systems that perform well on paper but fail to honour real people will never deliver health equity. Instead, we need maternal health leadership that is relational, responsive and rooted in justice. In practice, this means leaders should:

▸ Implement routine trauma-informed communication training across maternity teams.
▸ Ensure lived experience representation on governance boards, not solely advisory groups.
▸ Embed equity-related outcomes into performance dashboards alongside clinical metrics.
▸ Co-design service changes with women from communities most affected by poor outcomes, including remunerating their time.

Maternity care must honour the lived experiences of women in all their diversity and not reduce them to outcomes or efficiency targets. We must build and deliver care that is compassionate, dignified and trauma-informed. That means recognising how racism, ableism, poverty, migration status and other forms of structural exclusion, and importantly, how trauma shapes how care is received and experienced. An intersectional approach is essential because without it, we will continue to overlook the women most likely to be harmed by services that fail to reflect their needs.

This is not to say that data do not matter. Until all women are represented in the data, they cannot be counted. Maternity records should collect the data that help us understand who our services support, how they are experienced and how they affect outcomes. It is also telling that stillbirth, a profound and traumatic outcome of failed maternity systems, is not currently included in the Sustainable Development Goals or many national maternal health indicators. This omission reflects a deeper discomfort with acknowledging death and grief in maternal health narratives, but it must be addressed. The exclusion of stillbirth from key global targets is not just a technical gap, it is a moral one.^21^ Leadership in these spaces must insist on counting what matters, and what is currently being left out.

To care for women well, we also need to invest before crisis hits. That means investing across the continuum of women’s health from preconception health to the months and years beyond birth. We must also expand postpartum care beyond the traditional 6 to 12-week window. As emerging evidence shows, many women continue to experience physical, emotional and psychological effects of childbirth well into the first year and beyond.

Health leaders must also take up the mantle of advocacy. That means lobbying not only for improvements in services, but for sustained investment in maternal health research, as well as wider women’s health and rights. When women face health problems, whether in pregnancy or beyond, we need a robust, inclusive evidence base that reflects their lives and experiences. Investment is needed across the continuum of maternity care and beyond, from the conception of knowledge through high-quality intersectional research to the robust evaluation of new interventions and services designed to improve the care of mothers and their families.

One crucial maternal health priority is better support for prenatal health and early pregnancy loss. Almost everyone is touched by the heartbreak of miscarriage, if not personally, then through a friend, family member or colleague. In the UK alone, around 1 in 5 of pregnancies end in a loss during the first trimester, although this number is likely to be an underestimation. But beyond these numbers lies a deeply profound and complicated grief. The physical and emotional toll of early pregnancy loss can be as profound as any other bereavement. Partners feel this loss too, yet many families feel their tragedy is dismissed as a clinical episode, with little offered in the way of empathy or follow-up support. This lack of recognition within the health system and wider society only compounds the isolation and trauma of miscarriage, underscoring why compassionate prenatal and antenatal care, including care when pregnancy does not go as hoped, is so vital for maternal well-being.

Encouragingly, recent UK policy developments show a growing commitment to change. An independent Pregnancy Loss Review published in 2023 put forward 73 recommendations to improve care for parents experiencing baby loss before 24 weeks.^22^ It outlined a vision for consistent, compassionate support across the National Health Service (NHS). One outcome has been the introduction of a new pregnancy loss certificate in England; a voluntary scheme allowing parents to formally acknowledge and record the loss of a child prior to 24 weeks gestation. Another landmark change is the extension of statutory bereavement leave to cover miscarriages. Under reforms recently approved, mothers and partners will have a legal right to at least 1 week off work to grieve a loss before 24 weeks’ gestation.^23^ These developments send a powerful signal that early losses matter and that parents deserve time, space and recognition as they heal.

One growing solution for maternity care is for trauma-informed and culturally safe models to be embedded across all levels of care.^19^ Leaders must ensure maternity environments promote psychological safety and informed choice. We must also better equip staff, emotionally and practically, to handle complexity. Clinical training must include understanding trauma responses, working with diverse communities and supporting perinatal mental health needs.

We must also extend leadership beyond the walls of clinical care. That includes flexible working for staff with caregiving roles, meaningful staff involvement in service re-design, and stronger social care collaboration. Trauma-informed leadership applies as much to our teams as to our patients and their families. Creating environments of safety, autonomy and belonging matters for everyone.

## Leadership in the Mirror

Grief has a way of forcing you to confront who you are and what you stand for. In the mirror of my own loss, I have come to see the kind of leader I want to be. I am not the same doctor nor the same person I was before my daughter’s death. I lead differently now, with more empathy, more urgency and a clearer sense of purpose.

Leading differently means being changed by what we witness. It means having the humility to say, “we got this wrong” and the courage to ask, “what now?” I am still learning and always will be, but I know that silence will not serve us.

Leadership is not a destination. It is a discipline of reflection and reorientation. It means recognising when we have not done enough and then having the will to do better. For me, loss shattered the illusion that the system was safe, but it also lit the path to something better. I lead differently now. I have become more vocal in meetings, in research and in policy. I push for the inclusion of lived experience, to find those who are left in silence, not as a token gesture but as a structural requirement. I believe the care we provide reflects the leadership we embody. By sharing a small part of my own journey, I hope to encourage others in positions of power to examine themselves and to join in transforming maternity care through compassion, advocacy and justice.

This reflection contributes a lived-experience lens to leadership learning in maternity care. It demonstrates how personal grief can inform compassionate leadership and highlights systemic blind spots that leaders must address. Above all, it underscores that leadership grounded in humility and listening is essential for transforming maternity systems.

To lead in maternity care is to hold immense responsibility. It is not just about policies or plans. We must lead with care, with courage and with the clarity that women’s lives, and their children’s futures, deserve nothing less.

## Data Availability

Data sharing not applicable as no datasets generated and/or analysed for this study.
